# (2*SR*,4a*SR*,8a*SR*)-6-Oxoperhydro­naphthalene-2-carboxylic acid

**DOI:** 10.1107/S1600536808042700

**Published:** 2008-12-20

**Authors:** Georgia Efthimiopoulos, Mark Davison, Roger A. Lalancette, Hugh W. Thompson

**Affiliations:** a Carl A. Olson Memorial Laboratories, Department of Chemistry, Rutgers University, Newark, NJ 07102, USA

## Abstract

In the title racemic compound, C_11_H_16_O_3_, the mol­ecule adopts a conformation that places its carboxyl group in an equatorial position. Mol­ecules aggregate by hydrogen-bond pairing of carboxyl groups, yielding centrosymmetric dimers that are arranged into layers in the (020) planes.

## Related literature

For related structures, see: Efthimiopoulos *et al.* (2008 ▶); Lalancette *et al.* (2007 ▶). For other related literature, see: Borthwick (1980 ▶); Steiner (1997 ▶).
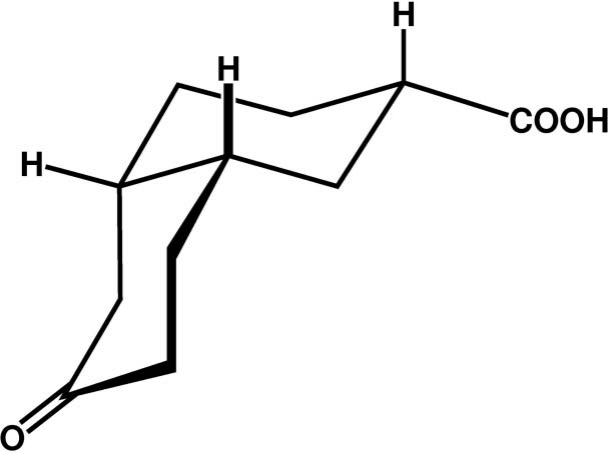


## Experimental

### 

#### Crystal data

C_11_H_16_O_3_
*M* *_r_* = 196.24
Monoclinic, 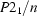

*a* = 5.3568 (1) Å
*b* = 22.3758 (2) Å
*c* = 8.3376 (1) Å
β = 99.593 (1)°
*V* = 985.39 (2) Å^3^
*Z* = 4
Cu *K*α radiation
μ = 0.78 mm^−1^
*T* = 100 (2) K
0.24 × 0.20 × 0.08 mm

#### Data collection

Bruker SMART APEXII CCD diffractometer
Absorption correction: multi-scan (*SADABS*; Sheldrick, 2008*a* ▶) *T*_min_ = 0.836, *T*_max_ = 0.941
8305 measured reflections
1675 independent reflections
1578 reflections with *I* > 2σ(*I*)
*R*_int_ = 0.019

#### Refinement

*R*[*F* ^2^ > 2σ(*F* ^2^)] = 0.034
*wR*(*F* ^2^) = 0.089
*S* = 1.05
1675 reflections
130 parameters
H atoms treated by a mixture of independent and constrained refinement
Δρ_max_ = 0.24 e Å^−3^
Δρ_min_ = −0.18 e Å^−3^

### 

Data collection: *APEX2* (Bruker, 2006 ▶); cell refinement: *SAINT* (Bruker, 2005 ▶); data reduction: *SAINT*; program(s) used to solve structure: *SHELXTL* (Sheldrick, 2008*b* ▶); program(s) used to refine structure: *SHELXTL*; molecular graphics: *SHELXTL*; software used to prepare material for publication: *SHELXTL*.

## Supplementary Material

Crystal structure: contains datablocks I, global. DOI: 10.1107/S1600536808042700/bi2325sup1.cif

Structure factors: contains datablocks I. DOI: 10.1107/S1600536808042700/bi2325Isup2.hkl

Additional supplementary materials:  crystallographic information; 3D view; checkCIF report

## Figures and Tables

**Table 1:** Hydrogen-bond geometry (Å, °)

*D*—H⋯*A*	*D*—H	H⋯*A*	*D*⋯*A*	*D*—H⋯*A*
O3—H3⋯O2^i^	0.85 (2)	1.81 (2)	2.6555 (13)	177.4 (18)

Symmetry code: (i) 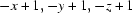.

**Fig. 1.**
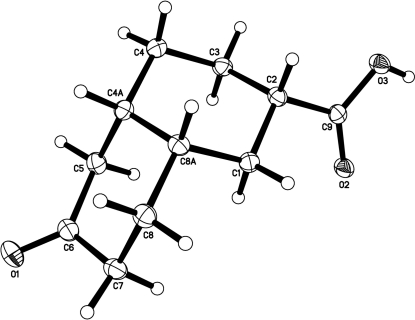
Molecular structure showing displacement ellipsoids at the 40% probability level for non-H atoms.

**Fig. 2.**
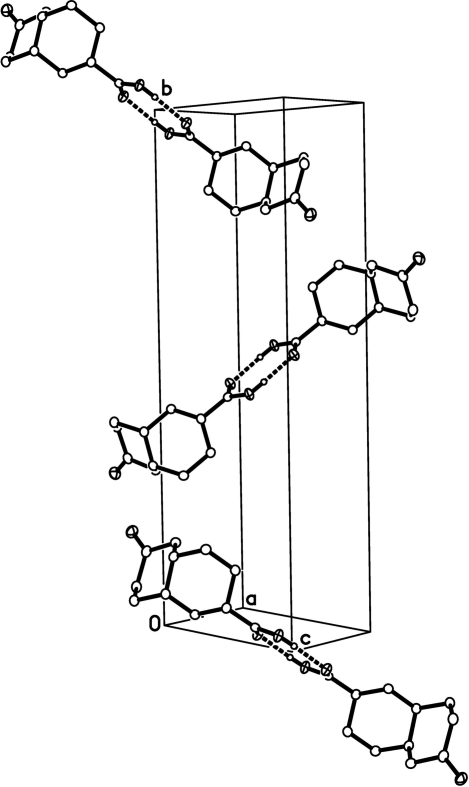
Partial packing diagram illustrating the pairing of molecules into centrosymmetric dimers centered at 1/2,1/2,1/2 and 0,0,0. All carbon-bound H atoms are omitted and displacement ellipsoids are drawn at the 40% probability level for non-H atoms.

## supplementary crystallographic information

### Comment

Our study of H-bonding modes in crystalline ketocarboxylic acids includes a variety of examples based on the naphthalene skeleton. Many of these are accessible from cyclohexanones *via* annulation reactions yielding enones, from which subsequent alkene reduction may then provide additional isoskeletal keto acids. The title racemate is the reduction product of an unsaturated keto acid whose structure we have previously reported (Efthimiopoulos *et al.*, 2008), and we have also reported the structure of the isoskeletal *trans* isomer (Lalancette *et al.*, 2007).

Fig. 1 shows the molecular structure and conformation. The unfavorable alternative conformer would place the carboxyl group at C2 not only on an axial bond, but inside the molecule's C-shaped curvature, while the observed conformer orients the carboxyl equatorially, with far less strain due to hindrance. This leaves as the only conformational option the rotational orientation of the carboxyl, which is turned so that the O2—C9—C2—C1 torsion angle is 31.57 (17) Å, presumably minimizing steric interactions with nearby H atoms at C1, C2 and C3. Although disorder-averaging of C—O bond lengths and C—C—O angles is common in carboxyl dimers, this is not observed in the current structure, where these values conform to ones typical for highly ordered cases (Borthwick, 1980).

Fig. 2 illustrates the packing of the chosen cell with centrosymmetrically hydrogen-bonded pairs of molecules; carboxyl dimers are centered at 1/2,1/2,1/2 and, in a second orientation, at 0,0,0. No intermolecular close contacts were found within the 2.6 Å range we standardly survey for C—H···O packing interactions (Steiner, 1997).

### Experimental

The title compound was synthesized from the isoskeletal unsaturated keto acid we have previously reported (Efthimiopoulos *et al.*, 2008), by room-temperature catalytic hydrogenation over 5% Pd/C in absolute EtOH at atmospheric pressure. This yielded the expected *cis* stereochemistry, and recrystallization from methyl pivalate gave material suitable for X-ray diffraction analysis (mp 399 K). The solid-state (KBr) infrared spectrum features a single broad peak at 1705 cm^-1^ for both C=O functions, typical of unstrained carboxyl-paired keto acids. In CHCl_3_ solution this combined absorption is seen at 1706 cm^-1^.

### Refinement

All H atoms were visible in Fourier difference maps. The position of the acid H was allowed to refine with its displacement parameter fixed at *U*_iso_(H) = 1.5*U*_eq_(O). The methylene and methine H atoms were placed in geometrically idealized positions and constrained to ride on their parent C atoms with C—H distances of 0.99 and 1.00 Å, respectively, and *U*_iso_(H) = 1.2*U*_eq_(C).

### Crystal data

**Table:**

C_11_H_16_O_3_	*F*(000) = 424
*M**_r_* = 196.24	*D*_x_ = 1.323 Mg m^−^^3^
Monoclinic, *P*2_1_/*n*	Melting point: 399 K
Hall symbol: -P 2yn	Cu *K*α radiation, λ = 1.54178 Å
*a* = 5.3568 (1) Å	Cell parameters from 6674 reflections
*b* = 22.3758 (2) Å	θ = 4.0–66.9°
*c* = 8.3376 (1) Å	µ = 0.78 mm^−^^1^
β = 99.593 (1)°	*T* = 100 K
*V* = 985.39 (2) Å^3^	Plate, colourless
*Z* = 4	0.24 × 0.20 × 0.08 mm

### Data collection

**Table:**

Bruker SMART APEXII CCD diffractometer	1675 independent reflections
Radiation source: fine-focus sealed tube	1578 reflections with *I* > 2σ(*I*)
graphite	*R*_int_ = 0.019
φ and ω scans	θ_max_ = 67.1°, θ_min_ = 4.0°
Absorption correction: multi-scan (*SADABS*; Sheldrick, 2008a)	*h* = −5→6
*T*_min_ = 0.836, *T*_max_ = 0.941	*k* = −26→25
8305 measured reflections	*l* = −9→9

### Refinement

**Table:**

Refinement on *F*^2^	Primary atom site location: structure-invariant direct methods
Least-squares matrix: full	Secondary atom site location: difference Fourier map
*R*[*F*^2^ > 2σ(*F*^2^)] = 0.034	Hydrogen site location: inferred from neighbouring sites
*wR*(*F*^2^) = 0.089	H atoms treated by a mixture of independent and constrained refinement
*S* = 1.05	*w* = 1/[σ^2^(*F*_o_^2^) + (0.0431*P*)^2^ + 0.4735*P*] where *P* = (*F*_o_^2^ + 2*F*_c_^2^)/3
1675 reflections	(Δ/σ)_max_ < 0.001
130 parameters	Δρ_max_ = 0.24 e Å^−^^3^
0 restraints	Δρ_min_ = −0.18 e Å^−^^3^

### Special details

**Table:**

Experimental. Crystal mounted on a Cryoloop using Paratone-N
Geometry. All e.s.d.'s (except the e.s.d. in the dihedral angle between two l.s. planes) are estimated using the full covariance matrix. The cell e.s.d.'s are taken into account individually in the estimation of e.s.d.'s in distances, angles and torsion angles; correlations between e.s.d.'s in cell parameters are only used when they are defined by crystal symmetry. An approximate (isotropic) treatment of cell e.s.d.'s is used for estimating e.s.d.'s involving l.s. planes.
Refinement. Refinement of *F*^2^ against ALL reflections. The weighted *R*-factor *wR* and goodness of fit *S* are based on *F*^2^, conventional *R*-factors *R* are based on *F*, with *F* set to zero for negative *F*^2^. The threshold expression of *F*^2^ > σ(*F*^2^) is used only for calculating *R*-factors(gt) *etc*. and is not relevant to the choice of reflections for refinement. *R*-factors based on *F*^2^ are statistically about twice as large as those based on *F*, and *R*-factors based on ALL data will be even larger.

### Fractional atomic coordinates and isotropic or equivalent isotropic displacement parameters (Å^2^)

**Table:**

	*x*	*y*	*z*	*U*_iso_*/*U*_eq_	
C1	1.0882 (2)	0.57235 (6)	0.84872 (15)	0.0191 (3)	
H1A	1.1496	0.5305	0.8541	0.023*	
H1B	0.9463	0.5749	0.9108	0.023*	
O1	1.1205 (2)	0.70766 (5)	1.31469 (12)	0.0309 (3)	
O2	0.62595 (18)	0.52821 (4)	0.67302 (11)	0.0234 (3)	
C2	0.9937 (2)	0.58935 (6)	0.67109 (15)	0.0188 (3)	
H2	1.1366	0.5844	0.6088	0.023*	
O3	0.76368 (19)	0.54230 (4)	0.43725 (11)	0.0234 (3)	
H3	0.636 (4)	0.5206 (8)	0.403 (2)	0.035*	
C3	0.9059 (3)	0.65526 (6)	0.65699 (15)	0.0204 (3)	
H3A	0.8545	0.6660	0.5410	0.024*	
H3B	0.7576	0.6606	0.7123	0.024*	
C4	1.1206 (3)	0.69626 (6)	0.73474 (16)	0.0214 (3)	
H4A	1.0582	0.7380	0.7307	0.026*	
H4B	1.2600	0.6943	0.6705	0.026*	
C4A	1.2244 (3)	0.67983 (6)	0.91180 (15)	0.0199 (3)	
H4A1	1.3801	0.7043	0.9468	0.024*	
C5	1.0342 (3)	0.69538 (6)	1.02598 (16)	0.0224 (3)	
H5A	1.0079	0.7392	1.0253	0.027*	
H5B	0.8694	0.6763	0.9842	0.027*	
C6	1.1230 (3)	0.67503 (6)	1.19831 (16)	0.0216 (3)	
C7	1.2161 (3)	0.61130 (6)	1.21789 (16)	0.0233 (3)	
H7A	1.0700	0.5837	1.1948	0.028*	
H7B	1.2991	0.6047	1.3318	0.028*	
C8	1.4047 (3)	0.59715 (6)	1.10296 (16)	0.0219 (3)	
H8A	1.5640	0.6193	1.1397	0.026*	
H8B	1.4443	0.5539	1.1091	0.026*	
C8A	1.3030 (2)	0.61380 (6)	0.92609 (15)	0.0189 (3)	
H8A1	1.4454	0.6086	0.8635	0.023*	
C9	0.7790 (2)	0.54990 (6)	0.59572 (15)	0.0182 (3)	

### Atomic displacement parameters (Å^2^)

**Table:**

	*U*^11^	*U*^22^	*U*^33^	*U*^12^	*U*^13^	*U*^23^
C1	0.0197 (7)	0.0186 (6)	0.0189 (7)	0.0004 (5)	0.0027 (5)	0.0005 (5)
O1	0.0357 (6)	0.0353 (6)	0.0219 (5)	0.0016 (4)	0.0059 (4)	−0.0084 (4)
O2	0.0235 (5)	0.0279 (5)	0.0191 (5)	−0.0076 (4)	0.0046 (4)	−0.0034 (4)
C2	0.0176 (7)	0.0214 (7)	0.0174 (6)	−0.0011 (5)	0.0036 (5)	−0.0015 (5)
O3	0.0223 (6)	0.0310 (5)	0.0162 (5)	−0.0079 (4)	0.0015 (4)	−0.0029 (4)
C3	0.0218 (7)	0.0214 (7)	0.0174 (6)	−0.0004 (5)	0.0020 (5)	0.0021 (5)
C4	0.0243 (7)	0.0196 (6)	0.0202 (7)	−0.0021 (5)	0.0035 (5)	0.0015 (5)
C4A	0.0197 (7)	0.0209 (7)	0.0188 (7)	−0.0042 (5)	0.0028 (5)	−0.0003 (5)
C5	0.0237 (7)	0.0215 (7)	0.0215 (7)	0.0015 (5)	0.0030 (5)	−0.0022 (5)
C6	0.0162 (7)	0.0288 (7)	0.0206 (7)	−0.0034 (5)	0.0053 (5)	−0.0034 (6)
C7	0.0241 (8)	0.0281 (7)	0.0174 (7)	−0.0009 (5)	0.0028 (5)	0.0015 (5)
C8	0.0195 (7)	0.0249 (7)	0.0206 (7)	0.0004 (5)	0.0011 (5)	0.0000 (5)
C8A	0.0160 (7)	0.0226 (7)	0.0185 (7)	−0.0001 (5)	0.0036 (5)	−0.0004 (5)
C9	0.0193 (7)	0.0168 (6)	0.0181 (6)	0.0034 (5)	0.0023 (5)	−0.0001 (5)

### Geometric parameters (Å, °)

**Table:**

C1—C2	1.5311 (17)	C4—H4B	0.990
C1—C8A	1.5336 (18)	C4A—C8A	1.5355 (18)
C1—H1A	0.990	C4A—C5	1.5454 (18)
C1—H1B	0.990	C4A—H4A1	1.000
O1—C6	1.2162 (16)	C5—C6	1.5066 (18)
O2—C9	1.2245 (16)	C5—H5A	0.990
C2—C9	1.5020 (18)	C5—H5B	0.990
C2—C3	1.5464 (18)	C6—C7	1.5107 (19)
C2—H2	1.000	C7—C8	1.5365 (18)
O3—C9	1.3210 (15)	C7—H7A	0.990
O3—H3	0.85 (2)	C7—H7B	0.990
C3—C4	1.5286 (18)	C8—C8A	1.5305 (17)
C3—H3A	0.990	C8—H8A	0.990
C3—H3B	0.990	C8—H8B	0.990
C4—C4A	1.5329 (18)	C8A—H8A1	1.000
C4—H4A	0.990		
			
C2—C1—C8A	111.14 (10)	C6—C5—C4A	112.57 (11)
C2—C1—H1A	109.4	C6—C5—H5A	109.1
C8A—C1—H1A	109.4	C4A—C5—H5A	109.1
C2—C1—H1B	109.4	C6—C5—H5B	109.1
C8A—C1—H1B	109.4	C4A—C5—H5B	109.1
H1A—C1—H1B	108.0	H5A—C5—H5B	107.8
C9—C2—C1	111.44 (10)	O1—C6—C5	122.40 (13)
C9—C2—C3	109.07 (10)	O1—C6—C7	121.85 (12)
C1—C2—C3	110.96 (10)	C5—C6—C7	115.74 (11)
C9—C2—H2	108.4	C6—C7—C8	111.51 (11)
C1—C2—H2	108.4	C6—C7—H7A	109.3
C3—C2—H2	108.4	C8—C7—H7A	109.3
C9—O3—H3	109.1 (12)	C6—C7—H7B	109.3
C4—C3—C2	110.02 (11)	C8—C7—H7B	109.3
C4—C3—H3A	109.7	H7A—C7—H7B	108.0
C2—C3—H3A	109.7	C8A—C8—C7	112.66 (11)
C4—C3—H3B	109.7	C8A—C8—H8A	109.1
C2—C3—H3B	109.7	C7—C8—H8A	109.1
H3A—C3—H3B	108.2	C8A—C8—H8B	109.1
C3—C4—C4A	113.01 (11)	C7—C8—H8B	109.1
C3—C4—H4A	109.0	H8A—C8—H8B	107.8
C4A—C4—H4A	109.0	C8—C8A—C1	112.32 (11)
C3—C4—H4B	109.0	C8—C8A—C4A	110.97 (10)
C4A—C4—H4B	109.0	C1—C8A—C4A	111.87 (10)
H4A—C4—H4B	107.8	C8—C8A—H8A1	107.1
C4—C4A—C8A	110.87 (10)	C1—C8A—H8A1	107.1
C4—C4A—C5	111.65 (11)	C4A—C8A—H8A1	107.1
C8A—C4A—C5	111.66 (10)	O2—C9—O3	122.68 (12)
C4—C4A—H4A1	107.5	O2—C9—C2	123.08 (11)
C8A—C4A—H4A1	107.5	O3—C9—C2	114.19 (11)
C5—C4A—H4A1	107.5		
			
C8A—C1—C2—C9	−178.60 (10)	C6—C7—C8—C8A	−51.60 (15)
C8A—C1—C2—C3	−56.84 (14)	C7—C8—C8A—C1	−70.54 (14)
C9—C2—C3—C4	179.35 (10)	C7—C8—C8A—C4A	55.51 (15)
C1—C2—C3—C4	56.23 (14)	C2—C1—C8A—C8	−178.96 (11)
C2—C3—C4—C4A	−55.28 (14)	C2—C1—C8A—C4A	55.48 (14)
C3—C4—C4A—C8A	53.90 (15)	C4—C4A—C8A—C8	−179.55 (11)
C3—C4—C4A—C5	−71.29 (14)	C5—C4A—C8A—C8	−54.37 (14)
C4—C4A—C5—C6	175.30 (11)	C4—C4A—C8A—C1	−53.25 (14)
C8A—C4A—C5—C6	50.55 (15)	C5—C4A—C8A—C1	71.94 (13)
C4A—C5—C6—O1	131.55 (13)	C1—C2—C9—O2	31.57 (17)
C4A—C5—C6—C7	−48.38 (16)	C3—C2—C9—O2	−91.28 (14)
O1—C6—C7—C8	−131.49 (13)	C1—C2—C9—O3	−150.80 (11)
C5—C6—C7—C8	48.44 (16)	C3—C2—C9—O3	86.36 (13)

### Hydrogen-bond geometry (Å, °)

**Table:**

*D*—H···*A*	*D*—H	H···*A*	*D*···*A*	*D*—H···*A*
O3—H3···O2^i^	0.85 (2)	1.81 (2)	2.6555 (13)	177.4 (18)

Symmetry codes: (i) −*x*+1, −*y*+1, −*z*+1.
